# Management changes resulting from hospital accreditation^1^

**DOI:** 10.1590/1518-8345.1394.2851

**Published:** 2017-03-09

**Authors:** João Lucas Campos de Oliveira, Carmen Silvia Gabriel, Hosanna Pattrig Fertonani, Laura Misue Matsuda

**Affiliations:** 2 Doctoral student, Universidade Estadual de Maringá, Maringá, PR, Brazil. Assistant Professor, Colegiado de Enfermagem, Universidade Estadual do Oeste do Paraná, Cascavel, PR, Brazil.; 3 PhD, Professor, Escola de Enfermagem de Ribeirão Preto, Universidade de São Paulo, WHO Collaborating Centre for Nursing Research Development, Ribeirão Preto, SP, Brazil.; 4 PhD, Adjunct Professor, Departamento de Enfermagem, Universidade Estadual de Maringá, Maringá, PR, Brazil.; 5 PhD, Associate Professor, Departamento de Enfermagem, Universidade Estadual de Maringá, Maringá, PR, Brazil.

**Keywords:** Hospital Accreditation, Hospital Administration, Quality Management, Nursing

## Abstract

**Objective:**

to analyze managers and professionals' perceptions on the changes in hospital management deriving from accreditation.

**Method:**

descriptive study with qualitative approach. The participants were five hospital quality managers and 91 other professionals from a wide range of professional categories, hierarchical levels and activity areas at four hospitals in the South of Brazil certified at different levels in the Brazilian accreditation system. They answered the question "Tell me about the management of this hospital before and after the Accreditation". The data were recorded, fully transcribed and transported to the software ATLAS.ti, version 7.1 for access and management. Then, thematic content analysis was applied within the reference framework of Avedis Donabedian's Evaluation in Health.

**Results:**

one large family was apprehended, called "Management Changes Resulting from the Accreditation: perspectives of managers and professionals" and five codes, related to the management changes in the operational, structural, financial and cost; top hospital management and quality management domains.

**Conclusion:**

the management changes in the hospital organizations resulting from the Accreditation were broad, multifaceted and in line with the improvements of the service quality.

## Introduction

The scientific and technological advances, the market competitiveness and the clients' increasing requirements in the service sector have driven organizations, also in health, to incorporate the quality management philosophy in their strategic and systematic perspective^1^ ^-^ ^2^. In that sense, concerning the external assessment of the quality, the health institutions' management has evolved and developed tools for this purpose^2^ ^-^ ^3^; and what is known today as Accreditation has been widely acknowledge for its potential to enhance the qualification of care all over the world, and also as a potential competitive strategy in the global health sector^3^ ^-^ ^4^.

Accreditation is defined as a systematic, periodical, reserved and sometimes voluntary strategy, in which its methods, based on preset quality standards, permit assessing the health services that may result or not in some certification level^4^ ^-^ ^5^. Although welcome, the certification the Accreditation grants is not the primary goal of this system, as the institutions that adhere to it should consider the continuous improvement and enhancement of the quality culture as priorities^6^.

In Brazil and other countries, the Accreditation inherited a strong North American influence, as this innovative quality management strategy originated in the United States of America (USA) and Canada more than five decades ago. The most representative Accreditation entity in the international context is the *Joint Commission International* (JCI), headquartered in the state of Illinois, USA^5^.

Under international influence, in 1999, the National Accreditation Organization (ONA) was founded, the main entity responsible for maintaining the Brazilian Accreditation. Its assessment method for certification rests on three levels: *Accredited* ; *Fully Accredited;* and *Accredited with degree of Excellence* , representing a scale of increasing criteria the service assessed needs to comply with^7^ ^-^ ^8^.

In the international sphere, in the hospital context, Accreditation has come with important benefits for the quality of care, such as: lower mortality rates at hospitals fully accredited by the system^9^; promotion of the culture and systematic use of quality tools^6^; satisfaction at work; strengthening of the multidisciplinary team; positive standards of compliance with indicators related to adverse events; better management of cerebrovascular accidents; user-centered care; respect for user rights, among others^7^ ^,^ ^10^ ^-^ ^12^.

In contrast with the above, although the number of studies on Accreditation seems to increase in the global universe of scientific publications^13^ in the main online database, published in Portuguese, English and Spanish, no studies were found that are specifically focused on the management changes deriving from the Accreditation process. This fact was verified in searches undertaken in the electronic libraries: *Biblioteca Virtual em Saúde* (BVS) and Scientific Electronic Library Online (SciELO); as well as in databases like the *Literatura Latino-Americana em Ciências da Saúde* (LILACS); National Library of Medicine (Pubmed); *Base de Dados em Enfermagem* (BDENF) and Scopus, concerning the period from 2004 until the start of the second half of 2014, using only the controlled descriptor "*Hospital Accreditation* " .

As the Accreditation is a system that seems to support strategies focused on quality in health, but new research is recommended on an international scale^13^, studies on management changes deriving from the Accreditation process are important and necessary because, based on their results, the managers of health institutions can (re)plan the management actions more assertively, focused on gaining certification and, mainly, on improving the services. Therefore, the following question is raised: Does the Accreditation process promote changes in hospital management? Of what kind? To answer these questions, the objective is to analyze the managers and professionals' perceptions on the hospital management changes deriving from the Accreditation.

## Method

Descriptive study with a qualitative approach, undertaken in May 2014, involving five hospital quality managers and 91 other professionals (n=96) working at four hospitals in the state of Paraná, Brazil. These places of study were accredited as follows in the Brazilian Accreditation procedure: Accredited Hospital; Fully Accredited Hospital; Accredited Hospital with the degree of Excellence; and Hospital that lost the certificate of Accreditation.

The places of study were selected in December 2013, in line with the following inclusion criteria: being a general hospital, located in the state of Paraná; possessing the Accreditation certificate (of each level) that has lasted a longer period, according to the validity of the certification available on the ONA website; or having lost its certificate (independently of the level) within a shorter period, according to the same website. Thus, three hospitals located in the state capital of Paraná were selected, and one (Fully Accredited) located in the metropolitan region of the same city, which was the only public organization.

Concerning the participants, it was established that the professionals responsible for the implementation and monitoring of the Accreditation, hereinafter called Quality Manager, would be the sole predetermined professional category for the data collection. The remaining professionals could relate to any hospital activity or sector, provided that they had participated at least in the most recent assessment for the sake of the revalidation of the Accreditation certificate. In view of this criterion, the sample was both convenience-based and intentional, based on the Quality Manager's indication, departing from the premise that that professional would know who participated more actively in the organization's external assessment. In both sampling procedures, the researchers sought to include different functions/activities performed at the hospitals.

All participants were contacted at their workplace, at a date and time they had personally arranged upon with the researcher. After making the appointment, the participants answered an individual, semi-structured interview that was guided by the following question: *"Tell me about the management of this hospital before and after the implementation of the Accreditation"* .

The researcher determined the total number of interviews when observing that the research objective had been reached. When the content of the interviews was successively repeated, that is, when testimonies were observed in distinct professional categories that transmitted a similar meaning and could be grouped. This procedure was followed at each hospital separately. The content of the interviews was fully transcribed and, then, the empirical material was transported to ATLAS.ti software, version 7.1, for the electronic management of the data.

After transferring the empirical material to the software mentioned, the data were submitted to thematic content analysis, respecting the following phases: pre-analysis; exploration of the material and treatment of the data^14^. Therefore, the software chosen was used to make it easier to handle a large information volume for analysis.

The interpretive data analysis was combined with the use of the application as follows: first, the entire corpus transported to ATLAS.ti was read. After skimming the testimonies, they were again read to survey the central ideas of the data^14^. Using a new analytic procedure mediated by repeated reading, the central ideas were grouped into cores of meaning or subcategories^14^ which, in the tool used, are interpreted as codes.

For each code, the representative statements, called quotations, were electronically selected and placed and numbered in each reference code. The final analysis was accomplished by clustering all emerging codes in a single thematic category called family. Two researchers consulted the entire material several times to clarify whether the selected quotations were related to the purpose of their respective codes.

The quotations most representative of each code were chosen as, due to the large data volume, demonstrating many excerpts of the participants' discourse would be unfeasible. In addition, in the presentation of the results, the excerpts/quotations of the reports were edited (or terms were added between brackets) to correct for possible grammatical errors, but without changing the essence.

In the ranking of the data, the semantic criterion was adopted, in which, as described, the messages the subjects issued are joined in categories and subcategories, according to the similarity among topics/themes^(14)^. The analysis and categorization were based on Donabedian's reference framework of Health Assessment, which systemized service assessment in the Structure (relatively stable elements in the organization); Process (the "doing" in health, which can be compared with what is established in the current norms); and Outcomes (the consequences of care and/or its absence/shortage for the user and the health organization, measured using tools, such as indicators)^15^.

It should be highlighted that, although the framework mentioned did not specifically need to picture what we intended to systemize in the presentation of the data, it was chosen because it was considered fundamental for the themes involving Assessment and Quality in Health, which are the actual core of the Accreditation^(3,7)^. Therefore, we departed from the premise that the choice of this theoretical support is essentially related to the research problem.

The participants were identified by the letter "I" (interviewee) and Arabic numerals indicating the chronological order of the interviews. The same was done for the hospital employer, in increasing order of certification (1, 2 and 3). Number four is related to the hospital that lost the certification.

All ethical and legal premises established in National Health Council Resolution 466/2012 were complied with and the research project was registered under CAEE: 28867014.7.0000.0104 and Opinion 623.509, issued by the Permanent Ethics Committee for Research Involving Human Beings at Universidade Estadual de Maringá (UEM), Maringá - PR. In addition, all participants read and signed the Free and Informed Consent Form, in two copies with the same content, which were also signed by the researcher.

## Results

The study involved 96 professionals, distributed as follows: 22 from the Accredited Hospital; 28 from the Fully Accredited Hospital; and 23 each from the Hospital Accredited with Excellence and the institution that lost its certificate. In total, five were Quality Managers as, in the second hospital, two professionals shared this function.

Among the other professionals (n=91), 69 (75.8%) were related to clinical service management or provision; 16 (17.6%) to administrative services; and six (6.6%) to hospital maintenance and support. Among the clinical professionals, nursing professionals (75.4%) played an important role. The participants' ages ranged between 22 and 58 years; women (78.2%) and married professionals (52%) were predominant.

Based on the content analysis of the interviews, one main family was defined using the software ATLAS.ti 7.1: "*Management changes resulting from hospital Accreditation: perspectives of managers and workers"* . This family clustered the content of five codes, according to the software.

**Figure 1:**
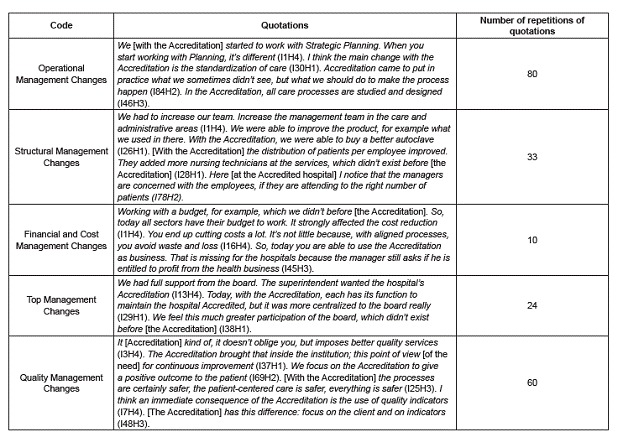
Codes related to the family *"Management changes resulting from hospital Accreditation: perspectives of managers and workers"* . Maringá, PR, Brazil, 2014

Due to the large information volume in the corpus, the results were systemized as follows (Figure 1).

## Discussion

Concerning the characteristics of the study sample, the large participation of nursing professionals and the predominance of women should be highlighted. It should be reminded that nursing is historically linked with care, the essence and purpose of the profession, which was also connected with the female figure in history. In addition, there is the fact that, in hospitals, nursing represents the largest group in the organizational human capital^16^.

The participants' testimonies revealed five subcategories or codes (Figure 1) related to the management changes deriving from the hospital Accreditation. Each element addressed in the codes and quotations, which were broad and aligned with what the Accreditation recommends, has a singular and relevant content. That is so because the content the managers and workers mentioned is related to the evaluation sections of the Brazilian Accreditation, particularly the first, which addresses the assessment of aspects related to the Management and Leadership of the institution assessed^8^.

Concerning the Operational Management Changes (80 quotations), one important element is the accomplishment of strategic planning in the organization, as appointed by I1H4. The relevance of this management action is weighted, because the execution of strategic planning is a fundamental management tool for services aiming for continuous quality improvement. Its actions support decision taking and further evaluation, in the form of a cyclical movement for the sake of quality as the desired product^1^ ^,^ ^17^.

In the context of the Operational Management Changes, the relation between the Accreditation and the mapping of care processes is emphasized. Thus, this is important and necessary because the Accreditation logic is based on the standardization of techniques and operational procedures, which can result in contributions to the safety of care delivery^6^ ^-^ ^7^ ^,^ ^11^. To give an example, a study developed in China evidenced that the standardization needed to achieve the JCI Accreditation promoted improvements in the drug prescriptions and administration^12^.

It is highlighted that the operational changes disseminated at the places of study are related to the Process dimension, according to the theoretical framework of Evaluation in Health^15^. That is so because the changes the participants mentioned indicate alterations in the work process which, influenced by the Accreditation, seems to have gained greater strategic impact in daily work.

In the context of the nursing services, the literature appoints that care process mapping can determine the time each work activity consumes; measure the workload; help to truly define the role of nursing professionals, especially nurses; redesign the work process; promote the qualification of actions and increase the productivity^18^.

Although the standardization of processes was considered an Operational Management Change, it also reflects management changes inherent in the Structure (33 quotations) of the Accredited hospital because the workload is directly related with the number of professionals working at the service/institution^19^. Thus, the quotations of I1H4, I28H1 and I78H2 refer to the increase in human capital in the hospital organization as a structural management change the Accreditation entails, a fact that is perfectly related with the first dimension of Donabedian's triad^15^ used to sustain the interpretation of the findings, as the Structure is considered as the valuation of the most "stable" aspects in the hospital organization, such as the collection of professionals, equipment, physical and financial structure.

It is important to increase the number of professionals as one of the management changes in the structure of hospitals that went through the Accreditation experience, considering that this can knowingly play a decisive role in the users' quality and safety. In that sense, especially in the hospital context, where nursing is the only group of professionals who monitor the users 24 hours per day, adequate dimensioning of the human capital is extremely important in terms of quality and quantity, because this can directly interfere in the results of the care process^19^.

The number of professionals to deliver safe care is not sufficient for qualified care, being widely disseminated at the moment that the leadership should also consider the quality of the staff a priority. Therefore, in another study developed in the South of Brazil, it was highlighted that the Accreditation was a determining factor to train the nurses for management activities^17^, which is one of the main sub-processes in these professionals' work and certainly gains intensity in the context of quality management systems. Nevertheless, ideas similar to the qualitative enhancement of the staff are not expressed in the quotations.

In the Brazilian Accreditation context, in which safety is the first evaluation level, adapting the number of nursing professionals to the patient care demand, as I28H1 mentions, in combination with the purchase of new technologies (I26H1), which advance exponentially in the health area, may mean reasserting this management system's social commitment to quality and safety in care^3^ ^,^ ^6^ ^-^ ^8^.

Another management change deriving from the Accreditation is related to the financial and cost management (10 quotations), in line with the Accreditation principles, because the relation between cost and quality is one element that has been discussed since the classical concepts of quality in the production of goods and services^2^ ^,^ ^15^. This assertion rests on the fact that, in health, efficiency is one of the pillars of quality, representing the measure of at what cost a given improvement in health is achieved^15^; therefore, the study findings may mean that, through well-defined management practices, the Accreditation essentially adopts the classical principles of quality in health.

In view of the above, it is noticed that some professionals legitimize the rationalization of cost as a management change deriving from the Accreditation, which may be related to the fact that, internationally, this strategy is considered an expensive process^20^. This assertion gains strength to the extent that two recent international literature reviews appoint that an important knowledge gap remains to clearly define the relation between cost and benefit of the Accreditation for the safety and quality of health services^13^ ^,^ ^21^.

Emphasizing cost management as a management change the Accreditation promotes may mean, beyond compliance with its principles, a way for the organization to survive in the market. This kind of actions can be observed in the quotation by I45H3, which signals the strategic potential the Accredited organization can reach. Thus, this can be interpreted as a change in the organizational Structure dimension, because it corresponds to the collection of the hospital capital^15^; however, due to the influence of the Accreditation, it is considered that the quality management system also influences the Process dimension^15^, as the changed structure seems to lever the work processes towards the corporate strategy.

It is highlighted that cost management is important in the Accreditation, also to favor corporate marketing, considering that the health sector is inserted in the competitive dynamics of service organizations; hence, some assert that Accreditation can position the health service as a promising business but that, therefore, the quality tools and cyclical assessment need to be used in a committed and systematic manner because, if not, the system can turn into increased bureaucracy for the institution^22^.

One way for the hospital to maintain a favorable relation between the cost-benefit of the services can be the adoption of statistic control methods of the care processes and products, guided by decisions that support the best measure between the cost and benefit to be achieved^2^ ^,^ ^15^. In these terms, top management decisions on hospital finances and costs will be necessary because, in Brazil, the hospitals that comply with the Accreditation are mainly private^23^ and, in these places, organizational cost decision are normally linked to the top management.

In line with the above, the quotations by E13H4, E29H1 and E38H1 should be considered in the code on top management changes (24 quotations), mentioning that the Accreditation process led to a change in the leadership style of the top management, changing from autocratic to participatory. This fact corresponds to the determinations of ONA as, particularly at the highest certification level, which is excellence in management, the decision process should not be centralized in the top management but shared with the team^8^.

The top management change the Accreditation entails can represent an important step for the organization to adhere to more participatory management action, which comprises the decentralization of decisions and the approximation among the members of the health team. This can interfere in the quality of the services^24^. Thus, in the light of the results, it is suggested that complying with participatory management principles can be yet another tool that, in combination with the Accreditation process, can add quality to the services.

Other contents were related to the code on quality management changes (60 quotations). Thus, the fact that the participants listed the change as resulting from the Accreditation may mean its social legitimation in the health context and the acknowledgement that this management system essentially seeks to advance the quality of the services, instead of being a mere supervision^6^ ^-^ ^8^.

The changes in quality management certainly influenced the change in the Process dimension of the organization because they interfere in the management practices with a view to improvements in care itself^15^. Nevertheless, it is suggested that these changes can also influence the Outcomes dimension^15^ as, if strategic and systematic management practices are used that are focused on the service quality, this can lever the performance of care outcomes, as the quotations from the code on this change illustrate, like the professionals who mentioned the enhanced safety of patients attended at the hospital that went through the Accreditation process.

All quotations from the code discussed are in line with the above, in view of the continuous improvement; user-centered care; care safety; systematic evaluation, like the use of indicators permits, all classical principles in the health area when it adopts quality management^7^ ^-^ ^8^.

Especially the cyclical assessment for the sake of user-centered improvements seems to affirm that the Accreditation complies with the classical principles of Health Assessment^2^ ^,^ ^15^. Thus, the Accreditation process tends to firmly establish itself as a quality management system or strategy in this singular production sector.

## Conclusion

These study results appoint that both the managers and workers acknowledge that the Accreditation entails management changes at the hospital, which happen in the following aspects: operational; structural; financial and cost; top hospital management and quality management. These management changes were unfolded into changes in the dimensions of Donabedian's triad (Structure, Process and Outcomes), used to clarify the findings.

In conclusion, the Accreditation resulted in broad and positive management changes in the hospital organizations, as the results indicated that the management practices at the investigated hospitals changed successfully and in different aspects, such as: standardization and mapping of care processes; enhancement of physical structure and work organization; improvements in hospital cost management; strategic positioning in the market; inclusion of participatory leadership, besides other initiatives that promote quality management.

The main limitation in this study is that the interviews were punctual (cross-sectional) and held at a limited number of places. Nevertheless, the knowledge produced can support the decision making of managers targeting the Accreditation certificate. In addition, clarifying aspects related to the management changes in hospital organizations going through external assessment can imply the enhancement of modern and systematic management practices with a quality focus.

Finally, it should be mentioned that new studies on the Accreditation are needed, especially aimed at analyzing the impact of this system on the quality of care; user safety; users and professionals' satisfaction; and the cost-benefit of its use.
